# Fate and adaptive plasticity of heterogeneous resistant population of *Echinochloa colona* in response to glyphosate

**DOI:** 10.1038/s41598-021-94370-7

**Published:** 2021-07-21

**Authors:** Md Asaduzzaman, Eric Koetz, Hanwen Wu, Michael Hopwood, Adam Shephard

**Affiliations:** NSW Department of Primary Industries, Pine Gully Road, Wagga Wagga, NSW 2650 Australia

**Keywords:** Plant sciences, Plant ecology

## Abstract

Understanding the fate of heterogenous herbicide resistant weed populations in response to management practices can help towards overcoming the resistance issues. We selected one pair of susceptible (S) and resistant (R) phenotypes (2B21-R vs 2B21-S and 2B37-R vs 2B37-S) separately from two glyphosate resistant heterogeneous populations (2B21 and 2B37) of *Echinochloa colona* and their fate and adaptive plasticity were evaluated after glyphosate application. Our study revealed the glyphosate concentration required to cause a 50% plant mortality (LD_50_) was 1187, 200, 3064, and 192 g a. e. ha^−1^ for the four phenotypes 2B21-R, 2B21-S, 2B37-R, and 2B37-S respectively. Both S phenotypes accumulated more biomass than the R phenotypes at the lower application rates (34 and 67.5 g a. e. ha^−1^) of glyphosate. However, the R phenotypes generally produced more biomass at rates of glyphosate higher than 100 g a. e. ha^−1^ throughout the growth period. Plants from the R phenotypes of 2B21 and 2B37 generated 32% and 38% fewer spikes plant^−1^ than their respective S counterparts in the absence of glyphosate respectively. The spike and seed numbers plant^-1^ significantly higher in R than S phenotypes at increased rates of glyphosate and these relationships were significant. Our research suggests that glyphosate-resistant *E. colona* plants will be less fit than susceptible plants (from the same population) in the absence of glyphosate. But in the presence of glyphosate, the R plants may eventually dominate in the field. The use of glyphosate is widespread in field, would favour the selection towards resistant individuals.

## Introduction

Herbicides have been widely used as an effective weed management tool since their discovery in the 1940s. However, this system often exerts strong selection pressure for resistance in weeds^1–3^, traits that is an inherited ability of a weed plant to survive an application of herbicide at its labelled use rate^4,5^. In this evolutionary process, both survival and reproduction of individuals with resistance alleles in a population are enriched in the presence of the herbicide^6^. Additionally, the dynamics and enrichment rate of resistance alleles are influenced by genetic (gene mutation rate, dominance, additivity, pleiotrophy, inheritance mode and ploidy), and biological factors (reproduction and mating system, population size and number of generations)^1,7–11^, as well as environmental conditions^7,9^. These factors can accelerate the herbicides selection pressure enabling weeds to withstand herbicide application^7,10^. It is believed that overreliance on herbicides, especially those belonging to the same chemical class or site of action is a major contributor of resistance seelction^12^.

Best management practices including knowledge about biology, ecology and fitness of suspected species can help overcome herbicide resistance^13^. Fitness is the impact of a herbicide-resistant allele on the survival and/or reproduction of resistant plants. Such fitness integrates all of the genetic, biochemical and physiological changes driven by a particular resistance gene^11,14,15^. Fitness is determined within an environment and is influenced by the success of other phenotypes that exist in the same population^16,17^. Determination of the fitness and adjustment ability of R phenotypes within a heterogenous populations is important for tackling ongoing herbicide resistance issues^15^. Because variation in underlying resistance may be transient and at non-equilibrium level but the population can eventually be shifted themselves to a completely defended state^18,19^. A proper quantification of the fate of a heterogenous natural population of weeds, helps predict the frequency of resistant (R) and susceptible (S) plants under various environmental conditions particularly under high selection pressures of herbicide. Also, the estimation of both survival and fecundity rates in resistant populations after herbicide exposure are a true ecological measure of resistance^19–21^.

Bioassays using isogenic lines, followed by estimation and comparison of differential fitness of both resistant and susceptible plants within the same population, can reduce the effect of genetic background^22^. However, most past studies have generally compared the fitness of resistant and susceptible plants from very different and geographically separate populations^23–26^. Of these, some compared many resistant populations with only one susceptible population^26–28^. It is important to evaluate R and S phenotype individuals from the same population^1^. In this experiment we used paired R and S lines of *Echinochloa colona* (awnless barnyard grass) selected from the same seed source collected from the same field to determine whether there were any fitness costs or gains associated with glyphosate resistance through estimation of their fate after glyphosate application. The inherent differences in genetic backgrounds between the populations could distort the results. The use of paired R and S lines in this study will overcome this issue.

*Echinochloa colona* is a self-pollinating annual grass generally considered to be naturalised and has exhibited resistance to glyphosate in Australia^29^. The fitness trajectory of resistance to glyphosate in *E. colona* has been investigated in a population in Western Australia^30^. But no information is available for populations from the Northern cotton cropping system of Australia, where glyphosate-based cropping systems are dominant and many glyphosate resistant populations of *E. colona* have been found. The information from Western Australia can be useful but the ecological zone and farming system is different from other parts of Australia.

Our study primarily confirmed glyphosate resistance in several populations of *E. colona* sourced from the Northern cotton cropping systems of Australia and evaluated the fitness of two separate pairs of R and S (resistant R versus susceptible S ) through estimation of survival and reproductive rates. Also, we observed different phenological traits of *E. colona* to determine if resistant populations exhibit a fitness benefit or cost through their phenological traits in addition to survival and reproduction (% seed production control).

## Results

### Preliminary screening

Preliminary resistance screening showed that four populations had the highest levels of resistance to glyphosate among the 18 tested populations (Table 1), with 2B21 being more resistant. A total of two resistant heterogenous populations (2B21 and 2B37) were selected for subsequent studies. Population 2B28 did not have enough seeds and therefore was excluded from the selection list.

**Table 1:**
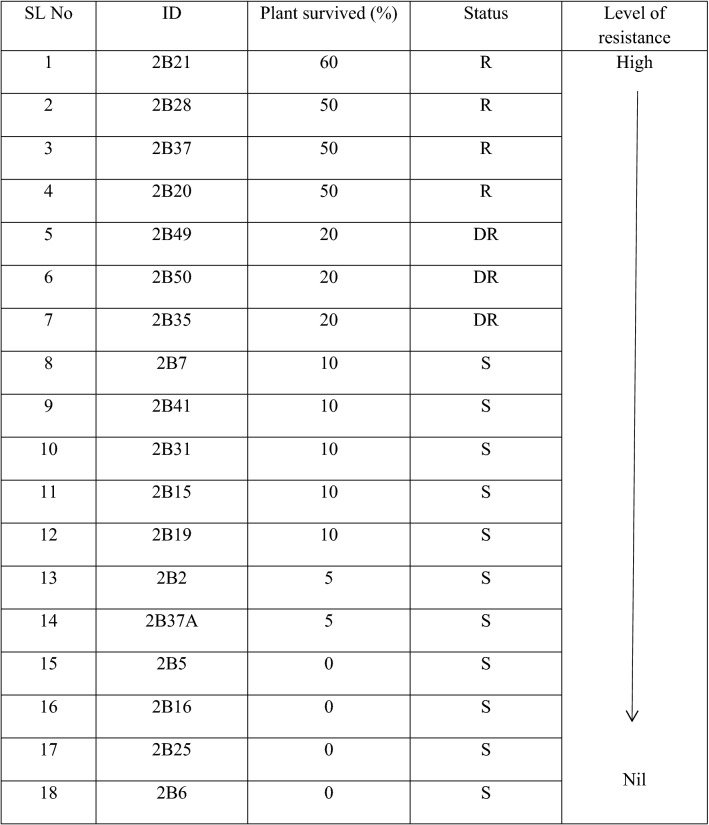
Populations of *E. conola* (awnless barnyard grass) are ranked based on plant survived (%) after application of glyphosate at 1350 g a. e. ha^−1^ *.

R, resistant; DR, developing resistant; S, susceptible.

### Selection of glyphosate-susceptible (S) and -resistant (R) paired lines of *E. colona*

When exposed to the glyphosate rate 540 g a. e. ha^−1^, more than 80% and 87% plants of R phenotypes of 2B21 and 2B37 survived respectively. We observed that all individual plants for both S and R phenotypes showed a prostrate growth form.

### Plant survival (%)

**Figure 1:**
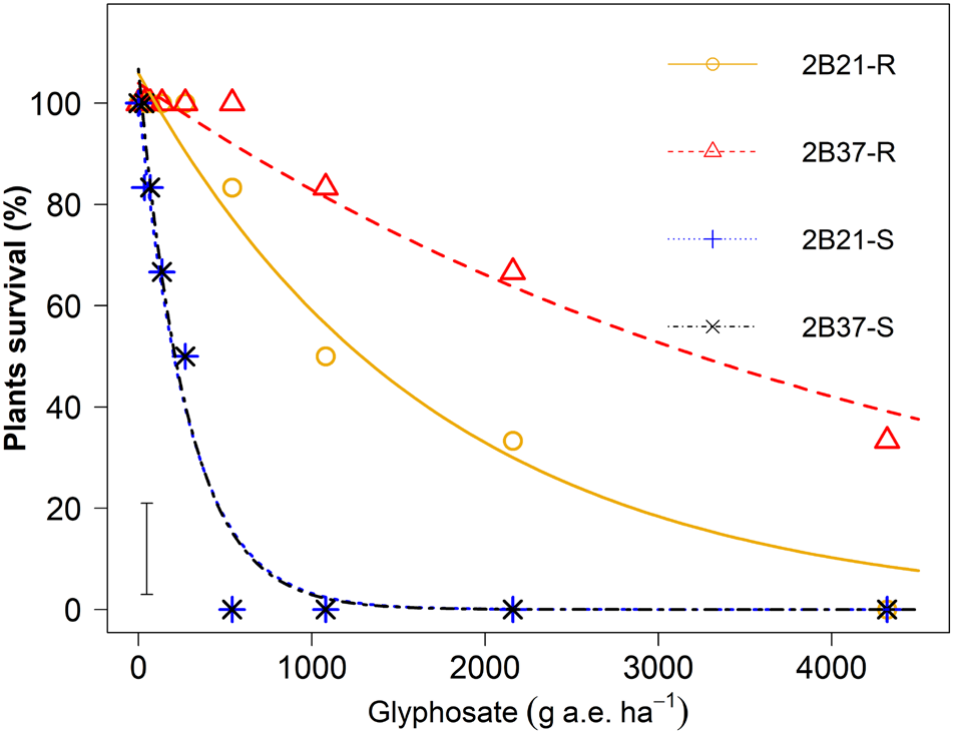
Plant survival (%) as a function of increasing glyphosate rates in glyphosate -susceptible (S) and -resistant (R) phenotypes derived from the two heterogeneous populations (2B21 and 2B37). The vertical bar represents the LSD _*p *< *0.05*_ value for differences between phenotypes.

There were significant (*p* < 0.001) differences in survival rates between the selected S and R phenotypes within populations (Fig. 1). Both susceptible phenotypes 2B21-S and 2B37-S were completely killed by glyphosate at 540 g a. e. ha^−1^. The LD_50_ value for 2B21-S and 2B37-S was 200 and 192 g a. e. ha^−1^ respectively and were significantly (*p* < 0.001) lower than the LD_50_ values of 1187 and 3064 g a. e. ha^−1^ calculated for corresponding resistant phenotype respectively (Table 2). Hence, glyphosate at 540 g a. e. ha^−1^ had little effect on the two R phenotypes 2B21-R and 2B37-R. By comparing the LD_50_ values of the four phenotypes, it was estimated that R phenotypes of both populations were more than fivefold resistant than their corresponding S phenotypes, and the R phenotype 2B37-R was 2.5-fold more resistant than the R phenotype of 2B21-R.

**Table 2:** Estimated regression parameters (Eq. 1) from glyphosate rate-response study on the basis of plant survival of the two heterogeneous populations (2B21 and 2B37) of *E. colona* containing S and R phenotypes.

Phenotype	*b*	Estimated LD_50_ (g a. e. ha^−1^)	± SE	RI
2B21-R	105.89	1187.78	232	5.90
2B21-S	100.81	200.92	42	–
2B37-R	103.95	3064.75	706	16
2B37-S	106.76	192.29	38	–

*b*, relative slope around *LD*_*50*_, where *LD*_*50*_ value is lethal or effective doses (g a. e. ha^−1^) causing 50% plant mortality. *SE* is standard error and *RI* is the Resistance Index.

### Above ground plant biomass (g)

**Figure 2:**
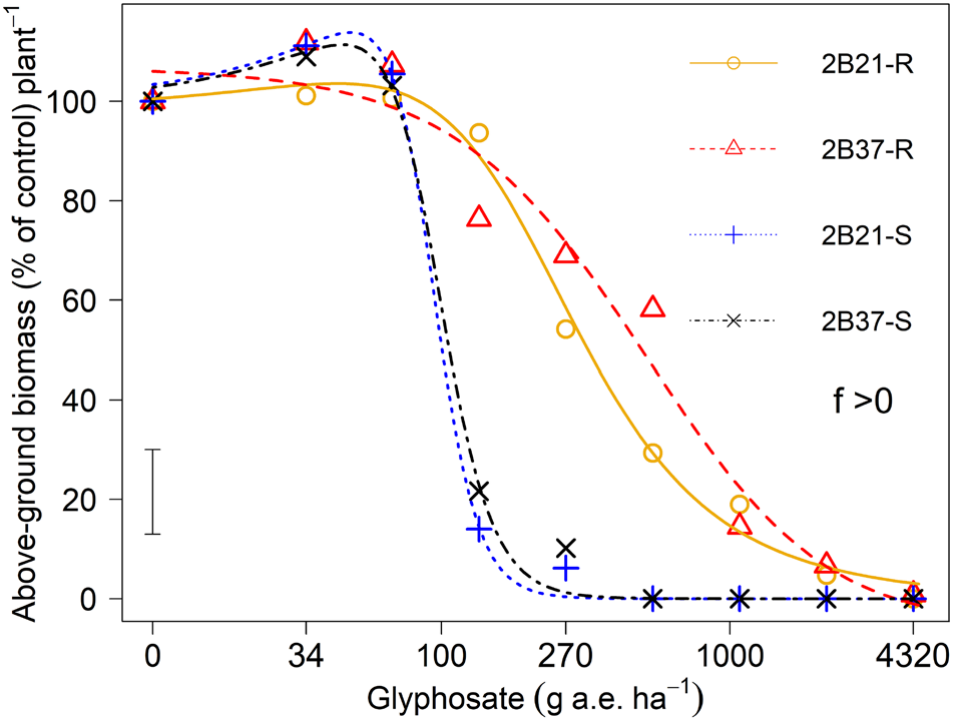
Aboveground biomass (% of control) of glyphosate-susceptible (S) and -resistant (R) phenotypes derived from two heterogeneous populations (2B21 and 2B37) of *E. colona*. The *f* > 0 indicates that there was a stimulation in S phenotypes with value of 0.35 and 0.33 for 2B21-S and 2B37-S respectively. The vertical bar represents the LSD _*p *< *0.05*_ value for differences between phenotypes.

There was a stimulation of aboveground biomass in both S phenotypes at 12 weeks after glyphosate application when the lower rate of glyphosate was used. The stimulation or hormetic dose range was between 34 and 67.5 g a. e. ha^−1^ of glyphosate (Fig. 2). About 10% stimulation occurred in both S phenotypes by glyphosate at 34 g a. e. ha^−1^. However, the hormesis ceased in both S phenotypes, and biomass production was reduced 80–90% when glyphosate rate was increased to more than 100 g a. e. ha^−1^. The biomass production of both S phenotypes was completely controlled (100%) at 540 g a. e. ha^−1^ of glyphosate application. Both R phenotypes maintained overall more biomass production than their S phenotypes throughout the life cycle except at 67.5 g a. e. ha^−1^ of glyphosate. The biomass inhibition of R phenotypes occurred as increased rate of glyphosate with LD_50_ value of 196 and 557 g a. e. ha^−1^ for 2B21-R and 2B37-R respectively.

### Number of spikes and seeds plant^-1^

**Figure 3:**
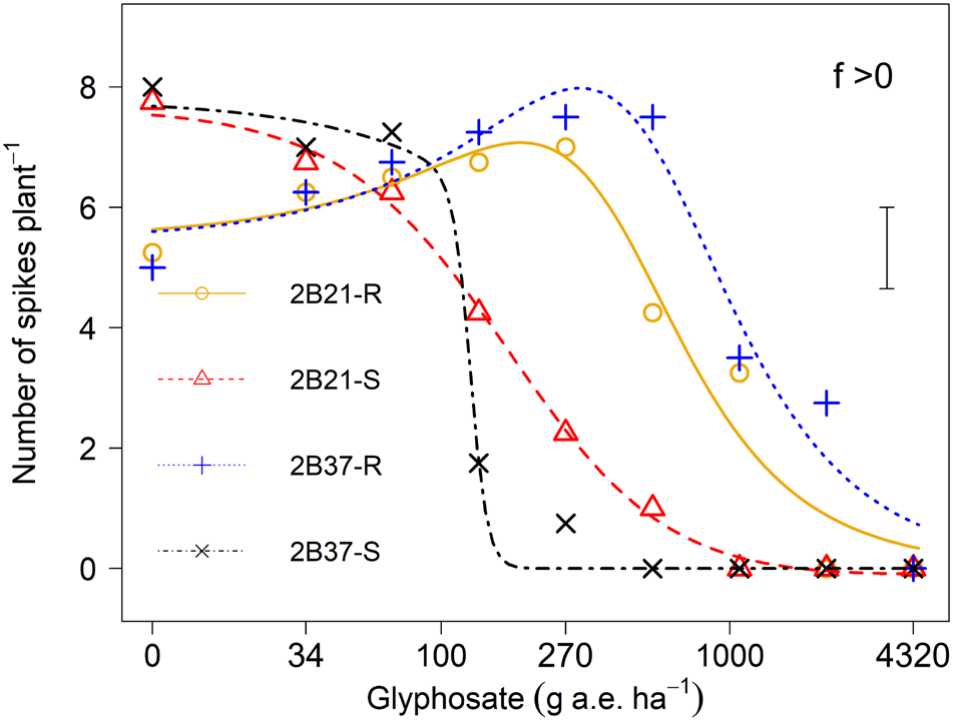
The number of spikes plants^-1^ in glyphosate-susceptible and -resistant phenotypes derived from two heterogeneous populations (2B21 and 2B37) of *E. colona*. The vertical bar represents the LSD _*p *< *0.05*_ value for differences between phenotypes.

**Figure 4:**
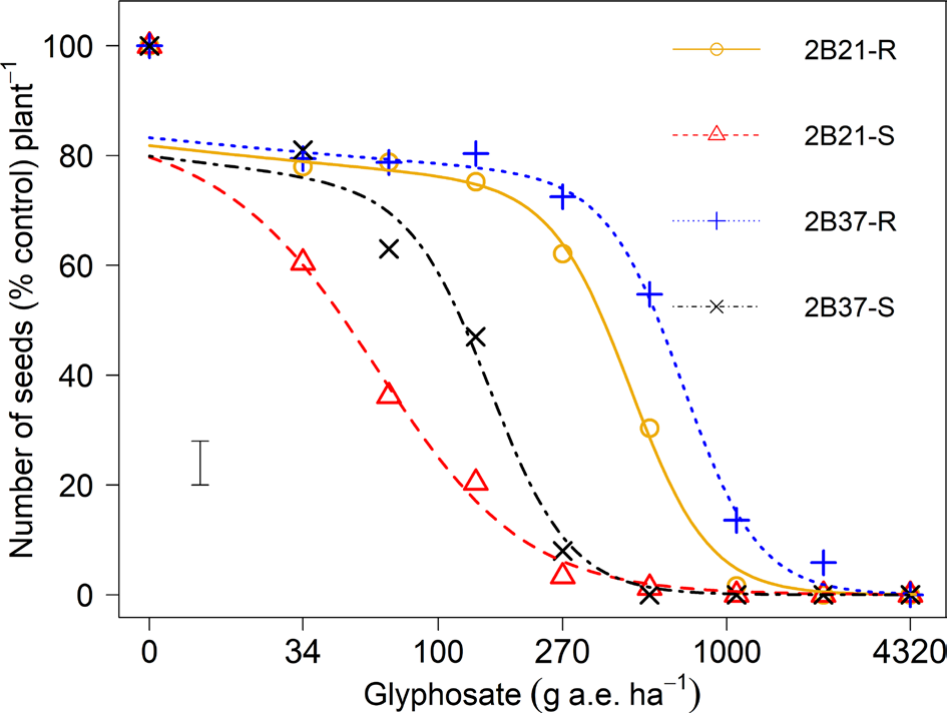
Seeds number (% mean of the control) in glyphosate-susceptible (S) and -resistant (R) phenotypes derived from the two heterogeneous populations (2B21 and 2B37) of *E. colona*. The vertical bar represents the LSD _*p *< *0.05*_ value for differences between phenotypes.

The plants of 2B21-R and 2B37-R generated 30 and 37% fewer spikes respectively than their susceptible individuals in the absence of herbicide respectively (Fig. 3). The spike numbers of S phenotypes were higher than R phenotypes at herbicide rates from 0 to 67.5 g a. e ha^−1^ but declined with increased rate of glyphosate. There was no spike formation in both S phenotypes when exposed to glyphosate at 540 g a. e. ha^−1^. Both R phenotypes produced more spikes than S phenotypes at glyphosate rates over 100 g a. e. ha^−1^. The seeds plant^-1^ declined with increasing herbicide rates in S and R phenotypes of both populations. Plant fecundity of the S and R phenotypes at the 540 g a. e. ha^−1^ glyphosate dose was quantified using the estimated equations from the regression model. Overall, the R lines produced more than twice the amount of the seeds than their corresponding S plants at each rate of glyphosate (Fig. 4).

### Spike formation and spikelets arrangement in spike

**Figure 5:**
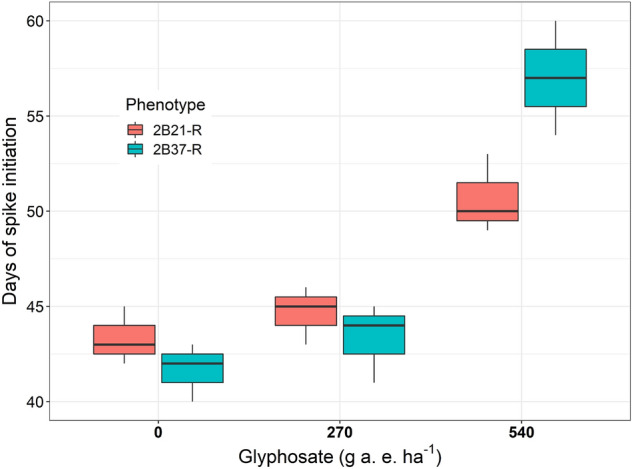
Box plots showing the required days to the first spike formation in glyphosate resistant phenotypes of two populations in the presence and absence of glyphosate. The centre of the boxes showing mean values and two extended vertical bars showing the range of the observed data points.

There was no spike formation in both S phenotypes after exposure to glyphosate at 540 g a. e. ha^−1^ or higher, therefore spike formation and spikelets arrangement in the spike were measured only in the R phenotypes. There was a significant (*p* < 0.001) effect of glyphosate rate (0, 270 and 540 g a. e. ha^−1^) to regulate the spike initiation in R phenotypes. Plants of the 2B21-R phenotype required 43 days for initiation of first spike in the absence of glyphosate. In comparison, the same phenotype took an additional 7 days when exposed to 540 g a. e. ha^−1^ glyphosate (Fig. 5). The plants from the 2B37-R phenotype took 41 and 57 days for spike initiation without and with the exposure to 540 g a. e. ha^−1^ glyphosate respectively.

**Figure 6:**
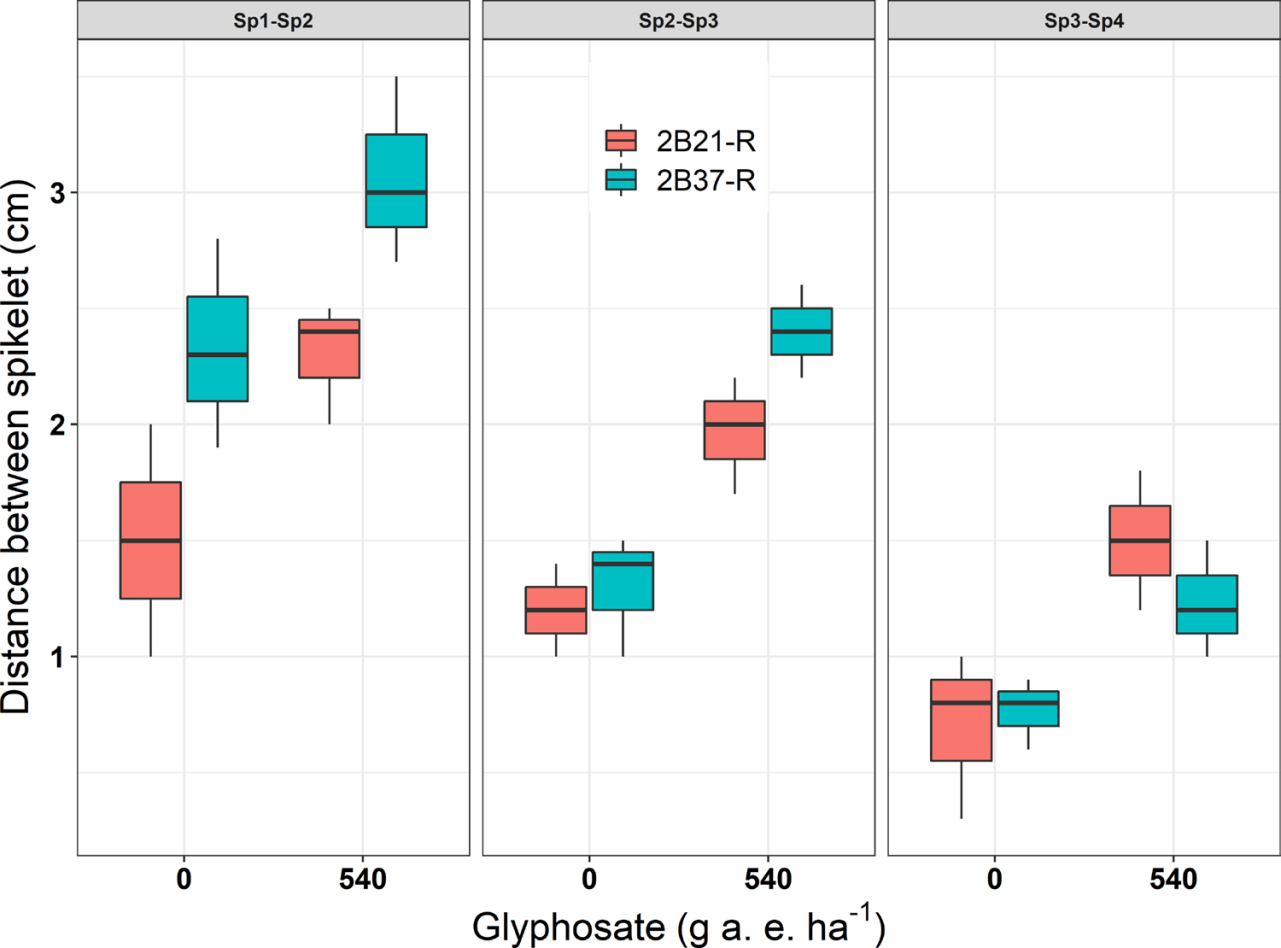
Box plots showing the distance (cm) among the spikelets within first appeared spike in glyphosate resistant phenotypes of two populations in the presence and absence of glyphosate. The centre of the boxes showing mean values and two extended vertical bars showing the range of the observed data points. Here Sp1-Sp2, Sp2-Sp3 and Sp3-Sp4 are the distance (cm) between first and second; second and third; and third and fourth spikelets respectively.

The distance between the first appeared spikelet (Sp1) and the second spikelet (Sp2), second and third (Sp3), and third and fourth (Sp4) was higher in glyphosate treated (540 g a. e. ha^−1^) than non-treated R plants (Fig. 6). For instance, the distance between Sp1 and Sp2 for 2B21-R are 1.2 cm and 2.3 cm in the absence and presence of glyphosate respectively. The distance between Sp1 and Sp2, and Sp2 and Sp3 was 2.3 cm and 3.1 cm for the 2B37-R without and with glyphosate application at 540 g a. e. ha^−1^ respectively.

### Fecundity

At the glyphosate rate of 540 g a. e. ha^−1^, the estimated survival rates of 2B21-S, 2B21-R, 2B37-S and 2B37-R phenotypes were 0, 83, 0 and 100% respectively (Table 3). Compared with plants not treated with glyphosate, seed production in the S plant was reduced as much as 100% at 540 g a. e. ha^−1^ glyphosate. However, the reduction of seed production for the R phenotype was 70% and 45% for phenotypes 2B21-R and 2B37-R respectively. This finding made it possible to estimate the fitness for both R phenotypes (W = 0.25 for 2B21-R and W = 0.55 for 2B37-R) relative to the fitness of these two phenotypes under no glyphosate treatment (W = 1) (Table 3). These results showed that 25% and 55% seeds would be returned to the soil seed bank for 2B21-R and 2B37-R respectively. At a lower glyphosate rate (270 g a. e. ha^−1^), higher numbers of S and R individuals survived (50 and 100%) compared to those treated with 540 g a. e. ha^−1^ of glyphosate. When the glyphosate dose doubled from 270 to 540 g a. e. ha^−1^, the fitness of the S plants was nil, and fitness decreased from 0.62 to 0.25 and from 0.74 to 0.55 for 2B21-R and 2B37-R phenotype respectively.

**Table 3:** Estimated fitness (W) for glyphosate-susceptible (S) and resistant (R) *E. colona* phenotypes based on survival rate and fecundity under glyphosate application of 270 and 540 g a. e. ha^−1^.

Glyphosate rate (g a. e. ha^−1^)	Phenotype	Survival rate	Fecundity	Fitness (W)
270	2B21-R	1	0.62	0.62
	2B21-S	0.5	0.03	0.02
	2B37-R	1	0.74	0.74
	2B37-S	0.5	0.08	0.04
540	2B21-R	0.8	0.30	0.25
	2B21-S	0	0	0
	2B37-R	1	0.55	0.55
	2B37-S	0	0	0

## Discussion

By using clonal lines derived from two confirmed heterogenous glyphosate resistant populations, we found that a high rate well beyond the label rate (576 g a. e. ha^−1^) was required to control both R phenotypes. The RI values for both R phenotypes indicate that glyphosate lost its inhibition potency for these two heterogeneous populations. There was a substantial variation among individual plants in the levels of glyphosate resistance within the two heterogeneous populations, resulting in the segregation of S and R phenotypes. The R phenotypes survival rate was higher compared with the S phenotypes at all glyphosate rates. Given the continued presence of glyphosate, the number of resistant individuals was likely to build up within the population over time. From a management perspective, we can assume that if there are more R plants present, the population will have more opportunity to produce R offspring than S. The role of such resistant transformations needs to be clarified for a wide range of geographically distributed populations of *E. colona.*

The different resistance factors between the R phenotypes might be due to different resistance mechanisms involved, which is an important area for further study. Differences in target site and non- target site resistance are commonly found in weeds^7,31–33^. The observed fitness costs emerged could be a side effect of the primary response to selection and manifest themselves differently on fitness. It is likely that this cost and adaptive plasticity may involve a rather numerous sets of epistatic interactions among resistance alleles, genetic background, and environment.

The S plants had vigorous early-season growth and produced more biomass than R phenotypes at low rates or in the absence of glyphosate. Our results are consistent with those of Schabenberger^34^: their dose–response study that analysed the hormesis effect of different herbicides. They observed that 5 g a. e. ha^−1^ glyphosate led to a 30% increase in dry weight of *E. crus-galli* (L.) P. Beauv^34^. However, S plants were unable to maintain such a trend with increasing rates of herbicide and R phenotypes produced more biomass than S phenotypes throughout their life cycle^34^. The increased biomass of S phenotypes at low rates of herbicide might help them to survive. The reproductive output of R plants at low rates of glyphosate indicated that a glyphosate resistant phenotype is the least-fit phenotype in the absence of glyphosate. But in the presence of glyphosate, it is likely that glyphosate resistant traits could be enriched in a population in a situation where applications of glyphosate are repeatedly used. Nevertheless, this type of life-cycle trade-off, is also likely to be species, mutation, and environment specific. Similarly, previous research reported that some herbicide-resistant species will be less fit than wild types in the absence of herbicide^35^.

The R phenotypes produced fewer spikes than the susceptible lines in the absence of glyphosate. This type of fitness cost has not been previously documented in *E. colona.* The magnitude of the fitness cost in our study indicates, in the absence of herbicide, a natural selection would tend to minimise levels of resistance. It is believed that species adaptive ability can be due to either individual phenotypic plasticity or intra-specific genetic differentiation to local environments^36–38^. For-instance, recently it was found that a low rate glyphosate-induced growth stimulation in *E. colona* where susceptible plants treated with glyphosate at a range of low rates (2.5 – 40 g a. e. ha^−1^) grew taller and produced more leaves, tillers, inflorescences and seeds than resistant plants^39^. Similarly, our data on the benefits of resistance in the presence of glyphosate suggest that there is likely to be strong positive selection for resistance in areas where glyphosate is sprayed. However, for areas where glyphosate is not sprayed, the costs of resistance that we measured suggest traits will be selected toward the S phenotype. Clearly, glyphosate use is increasing dramatically in Australia, hence other management practices are needed to be incorporated into an Integrated Weed Management (IWM) system for the existing glyphosate dependent cropping systems. The Australian cotton industry has developed the Herbicide Resistance Management Strategy (HRMS) to prolong the life of glyphosate in Roundup Ready® cotton. A multi-tactic or IWM weed management approach will help steward the ‘finite’ herbicide resources^40^. The HRMS still allows the use of glyphosate in combination with other tools to effectively disrupt target weeds. The use of soil-applied herbicides is one of the chemical options which would remove the seedlings that germinate early in the season and continue to provide residual control. Other options including inclusion of cover crops, and strategic tillage are suitable agronomic ways to enhance control of this weed species^41^^.^

The R phenotypes of *E. colona* plants took more time to form their first flower head at the recommended rate than the low rate of glyphosate. Additionally, the differences between spikelets within the spike were longer at the higher rates. It might be due to the metabolic disturbance caused by the altered shikimate pathway in plants^42^. Such changes come at a fitness cost in environments with high rates of herbicide, which eventually caused a stress to the plants and took more time for the stressed plants to initiate the spike with associated changes in the spikelets formation pattern. The R phenotypes of *E. colona* were known to be have high numbers of EPSPS gene copies and over time the stress can be diluted at a later growth stage in highly resistant plants^42^. This can be a trade-off for plant fitness and this trade-off was expected to be more obvious under stressed conditions. However, the underlying factors contributing to the delay in spike formation at a high rate of herbicide is yet to be fully understood.

The importance of a homogenous genetic background for the measurement of fitness costs has been argued^11^. It is likely that the magnitude and expression of fitness costs will vary between different genetic backgrounds, presenting the possibility that fitness costs will vary between populations and between individuals^43^. It also clarified that the genetics of weed population can be diverse and can influence the fitness penalty. Thus, the resistance testing with a single rate of herbicide on randomly selected plants from a heterogeneous natural population (seeds collected before grower applied herbicide) do not show a true picture of the resistant status in a population. It is important to use isogenic lines and evaluate their resistance level separately^22^. The resistance alleles may arise in a variety of genetic backgrounds and presence of genetic variation alone does not guarantee that resistance will evolve^44^. Research on fitness and gene flow process that link the disciplines of genetics and physiology with ecology is important to elucidate the population dynamics. A second prerequisite for the evolution of widespread resistance to glyphosate is the presence of net selection favouring increased resistance. The net selection acting on resistance is determined by both fitness costs and benefits^44^. Costs of resistance are the fitness reductions that are thought to arise from the diversion of limiting resources away from present and future growth and reproduction^9^. Benefits are the increases in fitness that result from the ability to reduce the detrimental effects on survival and reproductive success. Here, the longer distances between spikelets within spike, and the higher biomass production trend at high rates of glyphosate in R, might be the precise trade off to address the cost and benefits mechanism for resistant development and adaptive plasticity in R phenotypes.

## Conclusion

Management of herbicide resistance requires interdisciplinary approach to understand the mechanisms and dynamics of resistance. Fitness and gene flow can influence the evolution of and recovery from resistance in weed populations. Fitness costs as a result of the evolution of herbicide resistance in plants cannot be generalised. The fitness costs or evolutionary trade-offs associated with evolved resistance to herbicide are very diverse. Also, it is very likely that identification of fitness costs will vary, depending on weed species, herbicide (mode of action), the mechanism of resistance involved, and the genetic background through which resistance is expressed. Nevertheless, our study identified differences in fitness cost in several phenotypic traits where *E. colona* is a self-pollinating species. Plant fitness values of both S and R phenotypes differed under low versus high glyphosate rates. From a weed management viewpoint, the use of the recommended glyphosate field rate, combined with other IWM practices and regular resistance testing, is important to avoid a rapid increase of frequency of resistant phenotypes in the next generations. Also, the quantification of the selection intensity for resistance (i.e. relative R:S fitness under glyphosate selection) is an important parameter that needs to be considered for predicting the dynamics of glyphosate resistance alleles in agricultural production systems.

## Materials and methods

### Plant materials and preliminary screening

We selected a total of 18 populations from different cotton fields of Northern NSW and Southern Queensland of Australia. Prior to visit and seeds collection from these locations, relevant approvals were granted to address the farm biosecurity issues prior to seed collection. During seeds collection and experimental seeds were harvested from mature plants within a paddock and bulked as a population. Seeds (> 500) were sown in plastic pots (25 cm in diameter, pre-filled with potting mix) on 9 January 2017. We maintained the pots in a glasshouse at NSW Department of Primary Industries, Wagga Wagga (147°20′58.0 E, 35°03′09.1Ȳ″S). Seedlings were thinned to 10 plants/pot. Each population had 3 replications and a total of 30 plants. Seedlings at early-tillering stage were sprayed with a rate of glyphosate of 1350 g a. e. ha^−1^ on 10 February 2017. The herbicides were applied using an automated cabinet sprayer with a water volume of 77 L ha^−1^ using a flat fan nozzle at 300 kPa pressure. We assessed weed control ratings visually based on symptoms such as chlorosis, necrosis, stunting, and death of the treated plants at 28 days after treatment (DAT). Populations with mortality of around 90% after spraying with glyphosate were considered ‘‘susceptible,’’ whereas populations with more than 20% survival were considered as ‘‘resistant.’’^45^. The populations were ranked according to the survival rate and the two most resistant populations namely 2B21 and 2B37 were selected for subsequent experiments.

### Identification of glyphosate susceptible and resistant pairs from within population

**Figure 7:**
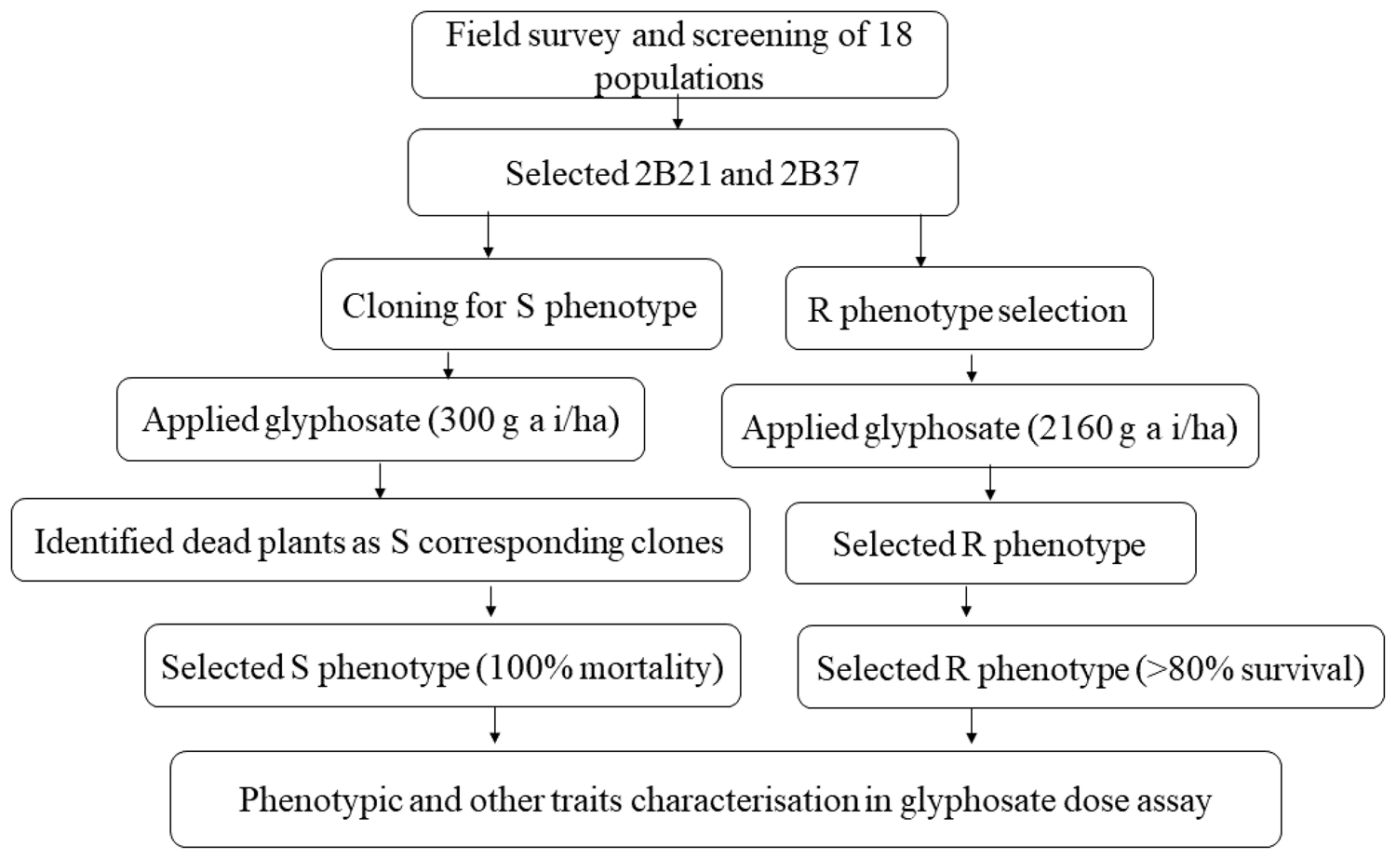
Experimental protocol for the identification of glyphosate-susceptible (S) and glyphosate-resistant (R) paired within the segregating glyphosate-resistant *E. colona* population.

We applied a modified plant cloning technique in the two selected populations (2B21 and 2B37) (Fig. 7)^30^. This approach was conducted under natural conditions at Wagga Wagga Agricultural Institute during the summer season in 2017. For the selection of R plants, sulphuric acid (98%) scarified seeds were germinated in plastic tray (35 × 30 cm) pre-filled with potting mix. Seedlings at the 2–3-leaf stage were treated with 2160 g a. e. ha^−1^ of glyphosate^30^. Plants were maintained outdoors after treatment and irrigated as required. We recorded plant survival up to 3 weeks after glyphosate treatment, and surviving plants were classified as R plants^30^. Those plants that appeared to be alive but lacked vigorous new growth were unclassified and discarded. For the selection of S plants, plants were cloned and numbered^30^. At the 3–4 tiller stage, seedlings were removed from the plastic trays and two tillers per plant (one clone) were excised^30^. These clones were trimmed to 1 cm of shoot material, re-potted and numbered accordingly. The ramet plants were transplanted with the same procedures. The clones at the 2–3-leaf stage was sprayed with 300 g a. e. ha^−1^ of glyphosate. Seedlings that did not survive the glyphosate treatment were classified as S plants. Glyphosate was applied in the same manner as previously mentioned. Identified S (from the untreated corresponding cloned plants) and R (from the treated surviving individuals) plants were individually transferred into bigger pots (25 cm in diameter and 28 in height) containing potting mix (Garden Essential, multi-purpose). We maintained a significant distance (> 500 m apart) between R and S individuals. Seeds from individual plants were harvested, cleaned, and stored in separate paper bags. The seeds dormancy was tested immediately after collection and different scarification methods were applied for dormant seeds collected from both R and S phenotypes.

### Growth and phenotypic characterisation of the paired R and S lines after exposure to glyphosate

Scarified seeds of the R and S pairs from both populations were sown on plastic trays (33 cm X 28 cm) containing potting mix. At 2–3 leaf stage, uniform seedlings of both R and S phenotypes were separately transplanted into plastic pots (25 cm in diameter and 28 in height). To minimise the effect of different plant densities on reproductive traits, one plant was transplanted per pot. The pots were kept outdoors. At 5–6 leaf stage, seedlings of both R and S phenotypes were treated with glyphosate at rates of 0, 34, 67.5, 135, 270, 540, 1080, 2160, and 4320 g a. e. ha^−1^, respectively. Pots were again placed outdoors after glyphosate application. Glyphosate effects on plant survival rates (% plant survived), above-ground biomass (g) and seed production (seeds/plant) were determined. Above-ground biomass of surviving plants for each glyphosate dose was harvested, dried at 60 ^∘^C for 72 h.

The phenotypic features included the date of first spike (Sp) formed, total spikes/plant, the distance between the first and second (Sp1-Sp2), second and third (Sp2-Sp3), and third and fourth (Sp3-Sp4) spikelets within a spike, visual scoring of seeds maturation time, and the total seed mass/plant was determined. Additionally, the weight of 300 seeds was quantified to estimate the total seed number/plant. Based on the estimated parameters of the non-linear regression model (see below), we calculated the amount of glyphosate to achieve 50% plant mortality (LD_50_), the above-ground biomass growth (GR_50_) and the seed yield (SY_50_) relative to the untreated control. Quantitative differences in glyphosate resistance level in terms of either survival, and biomass between the S and R phenotypes were calculated as a resistance index (RI) = LD_50_R/LD_50_S. The whole study complied with local, regional and research station’s regulations policy including demolished the plant and seeds material at a particular location to stop the accidental infestation of this species.

### Experimental design

We used a completely randomized design (CRD) for preliminary screening. The herbicide rate response study was conducted under a factorial design experiment, where herbicide rates and phenotypes had seven and four levels respectively. A way factorial design was considered for all the glyphosate dose response studies.

### Statistical analysis and model fitting

We analysed data using R software (R Core Team 2020) in RStudio (2020). Additionally, we used several R packages, including *drc*^46^, for explanatory data analysis. Data from the initial screening with a single rate of glyphosate were analysed by a binary logistic regression model. An exponential decay model (Eq. 1) was used for data from the survival study under different rates of glyphosate to estimate the glyphosate resistance parameters (LD_50_, GR_50_ SY_50_). The observed biomass, spikes/plant were fitted to a hormetic dose–response model proposed by Brain and Cousens^47^ (Eq. 2) and data from seeds plant^−1^ were fitted in hormetic dose–response model (Eq. 3)^48^

1 $$y = ae^{ - bx}$$

2 $$y = \left[ {c + \, (} \right[\left( {d - c} \right) \, + \, f x\left] {/ \, \{ 1 + \, exp \, } \right[b*logx\left( {x/e} \right)]\} )$$

3 $$y = \left[ {c + \, (} \right[\left( {d - c} \right) \, + \, f exp\left( { - 1/x^{a} } \right)\left] {/1 + \, exp \, } \right[b*logx\left( {x/e} \right)]\} )$$

In Eq. (1), *y* denotes the survival of plants at glyphosate rate *x*, *a* is the maximum plant response and *b* is the slope. In Eqs. (2) and (3), *d* denotes the mean response of the untreated control, *c* the mean response at infinite rates, *f* the degree of hormetic increase, *b* the slope of the decreasing curve part, *LD*_50_ the dose causing 50% mortality, and *e* parameter has no straightforward biological meaning^46^. The significance of hormesis was further cross-checked by an analysis of variance (ANOVA). The candidate models were assessed based on Akaike’s Information Criteria (AIC) and mean square root (MSE) values. In particular, the nested models were compared with MSE and non-nested models and were assessed based on the difference of AIC value (if the differences was > 2 then model with the lowest AIC was selected). The glyphosate rates resulting in 50% mortality (LD_50_) and fecundity (SY_50_) were estimated for both S and R phenotypes. SY_50_ was predicted using both total seeds mass (SY_m50_) and total seeds number (ST_m50_) per plant. Fitness (W) is a function of the proportion of plants that survived from seed dispersal to reproduction of the relative strength of selection for a R phenotype. Fitness of the S and R phenotypes of both populations were estimated after quantification of both the survival and fecundity rate at two glyphosate rates (270 and 540 g a. e. ha^−1^).
